# Activity of Cefiderocol Against *Enterobacterales*, *Pseudomonas aeruginosa*, and *Acinetobacter baumannii* Endemic to Medical Centers in New York City

**DOI:** 10.1089/mdr.2019.0298

**Published:** 2020-07-07

**Authors:** Alejandro Iregui, Zeb Khan, David Landman, John Quale

**Affiliations:** Division of Infectious Diseases, SUNY Downstate Medical Center, Brooklyn, New York, USA.

**Keywords:** Acinetobacter, Pseudomonas, Enterobacteriaceae

## Abstract

Therapeutic options for the treatment of infections owing to multidrug-resistant Gram-negative pathogens are often limited. Cefiderocol is a novel siderophore cephalosporin with activity against Gram-negative pathogens, including many multidrug-resistant strains. The activity of cefiderocol was examined against *Enterobacterales*, *Pseudomonas aeruginosa*, and *Acinetobacter baumannii* that included (1) a recent surveillance collection of clinical isolates, (2) a collection of carbapenem-resistant isolates from a previous surveillance study, and (3) a collection of well-characterized isolates. Susceptibility testing for cefiderocol was performed with iron-depleted cation-adjusted Mueller–Hinton broth. Cefiderocol minimum inhibitory concentrations (MICs) were correlated with resistance mechanisms in the well-characterized isolates. For the *Enterobacterales*, including a collection of KPC-possessing *Klebsiella pneumoniae*, cefiderocol MICs were all ≤4 mg/L. Cefiderocol MICs were two- to fourfold higher in cephalosporin-resistant isolates. For *K. pneumoniae*, MICs did not correlate with expression of genes encoding porins or efflux systems. For *P. aeruginosa*, >99% of isolates were inhibited by ≤4 mg/L, including the collection of carbapenem-resistant isolates. For *P. aeruginosa*, cefiderocol activity was not affected by expression of *ampC*, *oprD*, or several efflux systems. All the surveillance isolates of *A. baumannii*, and 88% of the collection of carbapenem-resistant isolates, had cefiderocol MICs ≤4 mg/L. MICs were twofold higher in *A. baummannii* isolates with proven extended-spectrum beta-lactamases, and cefiderocol activity did not correlate with expression of efflux systems. Cefiderocol demonstrated potent activity against important nosocomial pathogens. Continued development of this agent as a therapeutic option against multidrug-resistant bacteria should be encouraged.

## Introduction

The World Health Organization has declared antimicrobial resistance a global emergency and the development of new antimicrobials as a priority.^1^ Carbapenem-resistant *Enterobacterales*, *Pseudomonas aeruginosa*, and *Acinetobacter baumannii* are considered “critical” pathogens.^1^ The spread of multidrug-resistant Gram-negative pathogens has spurred the development of novel and potentially therapeutic agents. Next-generation aminoglycosides and β-lactamase inhibitors that have been recently brought into clinical practice are welcomed additions to our therapeutic armamentarium; however, resistance to these agents may limit their utility. For example, the diazabicyclooctanes are a new class of β-lactamase inhibitors that are active against many pathogens carrying serine β-lactamases.^2^ However, the currently available agents are not therapeutic options for isolates possessing metallo-β-lactamases. Clearly, alternative approaches are needed.

Cefiderocol (previously S-649266) is novel siderophore cephalosporin with a catechol moiety at position 3 of the cephalosporin side chain. This moiety facilitates formation of chelated complexes with iron and crosses the outer membrane of Gram-negative bacilli through active iron transporters.^3^ Several studies have shown potent *in vitro* activity of cefiderocol against *Enterobacterales*, *P. aeruginosa*, and *A. baumannii*, including isolates resistant to carbapenems.^4–8^ The spectrum of activity of cefiderocol includes isolates harboring a wide range of carbapenemases, including serine (KPC, OXA-type) and metallo (VIM, IMP, NDM) β-lactamases.^7–13^ Finally, in one clinical trial cefiderocol was comparable with imipenem–cilastatin for treatment of complicated urinary tract infections owing to carbapenem-susceptible pathogens.^14^

In this report we examine the *in vitro* activity of cefiderocol against *Enterobacterales*, *P. aeruginosa*, and *A. baumannii* endemic to New York City.

## Materials and Methods

### Isolates

Clinical isolates of *Enterobacterales*, *P. aeruginosa*, and *A. baumannii* underwent susceptibility testing. Three groups of *Enterobacterales* were analyzed, including (1) 2558 single-patient isolates of *Escherichia coli*, *Enterobacter* spp. (included in this group was *Klebsiella aerogenes*), and *Klebsiella pneumoniae* gathered during a 3-month surveillance study involving seven hospitals in Brooklyn, New York in 2017; (2) 111 carbapenem-resistant (and KPC-possessing) isolates of *K. pneumoniae* gathered from a similar surveillance study performed in 2013–2014; and (3) 34 well-characterized isolates of *K. pneumoniae*.^15–17^ For the last group, the presence of β-lactamases and genetic expression of *bla*_KPC_, *ompK35*, *ompK36*, and *acrB* were previously determined.^17^ Multilocus sequence typing was performed on select isolates of *K. pneumoniae* according to established protocols.^18^

For *P. aeruginosa* and *A. baumannii*, a similar three groups of isolates were analyzed. These groups included (1) 269 single-patient isolates of *P. aeruginosa* and 46 isolates of *A. baumannii* collected during the 2017 surveillance study, (2) carbapenem-resistant isolates of *P. aeruginosa* (*n* = 130) and *A. baumannii* (*n* = 78) gathered during a 2013–2014 surveillance study, and (3) 33 isolates of *P. aeruginosa* and 34 isolates of *A. baumannii* that were previously characterized for mechanisms of antimicrobial resistance.^19–21^ For *P. aeruginosa*, the presence of β-lactamases and genetic expression of *ampC*, *oprD*, *mexA*, *mexC*, *mexE*, and *mexX* were analyzed.^20^ For the *A. baumanii* isolates, the presence of β-lactamases and genetic expression of *ampC*, *oxa51*, *adeB*, and *abeM* were determined, as previously described.^21^

### Susceptibility testing

Cefiderocol minimum inhibitory concentrations (MICs) were performed in iron-depleted cation-adjusted Mueller–Hinton broth.^22^ MICs for the remaining antibiotics were performed by the agar dilution method with Mueller–Hinton agar according to established CLSI methods.^23^ Susceptibility rates were determined using CLSI criteria; for cefiderocol, the provisional breakpoint of 4 mg/L was used.^24^ Control strains included *E. coli* ATCC 25922 and 35218 and *P. aeruginosa* ATCC 27853.

Statistical analysis using the two-tailed Student's *t*-test and multiple linear regression were used to compare MICs with characterized mechanisms of resistance. A value of *p* < 0.05 was considered significant.

## Results

### Enterobacterales

Among the surveillance isolates gathered in 2017 (Table 1), all *E. coli* isolates (*n* = 1869) had cefiderocol MICs ≤2 mg/L. Among the ceftazidime-resistant isolates (*n* = 141), the MIC_50_/MIC_90_ values were 0.5/2 mg/L, which were fourfold higher compared with the values of the ceftazidime-susceptible isolates (0.12/0.5 mg/L). The mean cefiderocol MIC was higher in the group resistant to ceftazidime vs. the isolates susceptible to ceftazidime (0.55 ± 0.51 vs. 0.16 ± 0.17 mg/L, *p* < 0.001). Similarly, all the *Enterobacter* spp. (*n* = 172, including 58 isolates of *K. aerogenes* and 104 isolates of *Enterobacter cloacae*) had cefiderocol MICs ≤2 mg/L. For the *Enterobacter* isolates that were resistant to ceftazidime (*n* = 38), the MIC_50_/MIC_90_ values for cefiderocol were 0.25/1 mg/L, which were twofold higher than those values of the ceftazidime-susceptible isolates (0.12/0.5 mg/L). In addition, the 18 isolates of *Enterobacter* that were nonsusceptible to piperacillin/tazobactam (and presumably AmpC hyperproducers) had MIC_50_/MIC_90_ values for cefiderocol that were twofold higher than that of the susceptible isolates (0.25/1 vs. 0.12/0.5 mg/L, respectively).

**Table 1.** Susceptibility Results Involving Enterobacteriaceae from the 2017 Surveillance Collection and the 2013–2014 Carbapenem-Resistant Collection

	MIC_50_	MIC_90_	Range	
	mg/L			Susceptible, %
2017 surveillance isolates				
*Escherichia coli* (*n* = 1869)				
Cefiderocol	0.12	0.5	≤0.03 to 2	100
Piperacillin/tazobactam	2/4	4/4	≤0.25/4 to >128/4	99
Ceftriaxone	≤0.06	16	≤0.06 to >32	88
Ceftazidime	0.25	2	≤0.12 to >32	92
Meropenem	≤0.12	≤0.12	≤0.12 to 4	99.9
Gentamicin	1	>16	≤0.25 to >16	86
TMP/SMX	≤0.25/4.75	>4/76	≤0.25/4.75 to >4/76	64
Ciprofloxacin	≤0.12	>4	≤0.12 to >4	67
*Enterobacter* spp. (*n* = 172)				
Cefiderocol	0.12	0.5	≤0.03 to 1	100
Piperacillin/tazobactam	4/4	32/4	≤0.25/4 to >128/4	90
Ceftriaxone	0.25	32	≤0.06 to >32	81
Ceftazidime	0.5	32	≤0.12 to >32	83
Meropenem	≤0.12	≤0.12	≤0.12 to >8	98
Gentamicin	1	1	≤0.25 to >16	94
TMP/SMX	≤0.25/4.75	>4/76	≤0.25/4.75 to >4/76	84
Ciprofloxacin	≤0.12	1	≤0.12 to >4	91
*Klebsiella pneumoniae* (*n* = 517)				
Cefiderocol	0.12	0.5	≤0.03 to 2	100
Piperacillin/tazobactam	4/4	8/4	≤0.25/4 to >128/4	96
Ceftriaxone	≤0.06	>32	≤0.06 to >32	83
Ceftazidime	0.25	16	≤0.12 to >32	84
Meropenem	≤0.12	≤0.12	≤0.12 to >8	96
Gentamicin	0.5	8	≤0.25 to >16	89
TMP/SMX	≤0.25/4.75	>4/76	≤0.25/4.75 to >4/76	79
Ciprofloxacin	≤0.12	>4	≤0.12 to >4	85
2013–2014 Carbapenem-resistant surveillance isolates				
*K. pneumoniae* (*n* = 111)				
Cefiderocol	1	2	≤0.03 to 4	100

TMP/SMX, trimethoprim sulfamethoxazole.

All the 2017 surveillance isolates of *K. pneumoniae* (*n* = 517) had cefiderocol MICs of ≤2 mg/L, including 19 isolates with *bla*_KPC_. Of the 19 *bla*_KPC_-possessing isolates, 12 belonged to ST258, two belonged to ST340, and one each belonged to ST45, ST327, ST584, ST3359, and ST3369. Compared with the ceftazidime-susceptible isolates, the ceftazidime-resistant isolates (but lacking *bla*_KPC_) had greater mean cefiderocol MICs (0.43 ± 0.46 vs. 0.21 ± 0.20 mg/L, *p* < 0.001) and MIC_50_/MIC_90_ values (0.25/1 vs. 0.12/0.5 mg/L). For the 111 KPC-possessing isolates gathered in 2013–2014, the MIC_50_/MIC_90_ values for cefiderocol were 1 and 2 mg/L, and all had an MIC of ≤4 mg/L.

There were 34 previously characterized isolates of *K. pneumoniae*, including 14 with the carbapenemase KPC (Supplementary Table S1). The mean cefiderocol MIC for isolates (*n* = 10) that did not have an extended-spectrum beta-lactamase (ESBL) or KPC β-lactamase was 0.24 ± 0.18 mg/L (range = 0.06–0.5 mg/L). The mean cefiderocol MIC for isolates (*n* = 10) that possessed only an ESBL (*bla*_SHV_) was 1.1 mg/L ± 1.09 mg/L (range = 0.25–4 mg/L; *p* = 0.04 compared with isolates lacking an ESBL). The one isolate in this group with an MIC = 4 mg/L also possessed an AmpC-type (ACT-1) enzyme. Four isolates with *bla*_KPC_ but without an ESBL had cefiderocol MICs of 0.5–1 mg/L (mean 0.875 mg/L). The remaining 10 isolates possessed both an ESBL and KPC, with a mean cefiderocol MIC of 1.07 ± 1.19 mg/L (*p* = 0.07 compared with isolates lacking an ESBL, range = 0.06–4 mg/L). There was no correlation between cefiderocol MICs and expression of *bla*_KPC_, the efflux-related genes *marA*, *ramA*, *soxS*, and *acrB*, and the porin-related genes *ompK*35 or *ompK*36. Isolates with a frameshift mutation involving *ompK*35 had similar MICs as isolates without this mutation.

### *P. aeruginosa* and *A. baumannii*

#### Pseudomonas aeruginosa

There were 269 isolates of *P. aeruginosa* gathered in the 2017 surveillance study (Table 2), and 99.6% had a cefiderocol MIC ≤4 μg/mL. Compared with the isolates susceptible to ceftazidime, the nonsusceptible isolates had MIC_50_/MIC_90_ values for cefiderocol that were twofold higher (0.5/4 vs. 0.25/2 mg/L), but mean values were similar (0.84 ± 1.09 vs. 0.75 ± 1.15 mg/L, *p* = NS). There were 130 carbapenem-nonsusceptible isolates gathered in the 2013–2014 surveillance study, and the MIC_50_/MIC_90_ values were 0.5/1 mg/L.

**Table 2.** Susceptibility Results Involving *Pseudomonas aeruginosa* and *Acinetobacter baumannii* from the 2017 Surveillance Collection and the 2013–2014 Carbapenem-Resistant Collection of Isolates

	MIC_50_	MIC_90_	Range	
	mg/L			Susceptible, %
2017 surveillance isolates				
*P. aeruginosa* (*n* = 269)				
Cefiderocol	0.25	0.5	≤0.03 to 8	99.6
Piperacillin/tazobactam	8/4	128/4	2/4 to >128/4	75
Ceftazidime	4	32	1 to >32	83
Meropenem	1	8	≤0.12 to >8	76
Gentamicin	2	8	0.5 to >16	79
Ciprofloxacin	0.25	>4	≤0.12 to >4	69
2013–2014 Carbapenem-resistant surveillance isolates				
*P. aeruginosa* (*n* = 130)				
Cefiderocol	0.5	1	≤0.03 to 4	100
2017 surveillance isolates				
*A. baumannii* (*n* = 46)				
Cefiderocol	0.25	1	0.06 to 4	100
Piperacillin/tazobactam	32/4	>128/4	≤0.25/4 to >128/4	43
Ceftazidime	8	>32	≤0.12 to >32	54
Meropenem	4	8	≤0.12 to >8	48
Gentamicin	2	>16	0.5 to >16	70
Ciprofloxacin	>4	>4	≤0.12 to >4	46
2013–2014 Carbapenem-resistant surveillance isolates				
*A. baumannii* (*n* = 78)				
Cefiderocol	0.5	8	0.12 to >32	88

There were 33 characterized isolates of *P. aeruginosa* (Supplementary Table S2). Isolates with increased expression of *ampC* (>10 times control) had similar cefiderocol MICs compared with isolates without increased expression of this enzyme (0.67 ± 1.01 vs. 0.27 ± 0.23 mg/L, *p* = 0.2). By regression analysis, there was no correlation between cefiderocol MICS and expression of *ampC mexA*, *mexC*, *mexE*, *mexX*, and *oprD*.

#### Acinetobacter baumannii

There were 46 isolates of *A. baumannii* gathered in 2017, and all had cefiderocol MICs ≤4 mg/L. Compared with the isolates susceptible to ceftazidime, the MIC_50_ value for cefiderocol was twofold higher in the 18 ceftazidime-nonsusceptible isolates (0.25 vs. 0.12 mg/L). There were 78 carbapenem-resistant isolates gathered in 2013–2014, including 47 with *bla*_OXA-23-type_ β-lactamases. Overall, the MIC_50_/MIC_90_ values were 0.5/8 mg/L, which was identical to the subset of isolates with *bla*_OXA-23-type_ β-lactamases.

There were 34 isolates characterized of *A. baumannii* (Supplementary Table S3). Isolates with an SHV ESBL had significantly higher cefiderocol MICs than isolates without ESBLs (8.04 ± 11.58 vs. 0.86 ± 1.08 mg/L, *p* = 0.02). By regression analysis, there was no correlation between cefiderocol MICs and expression of genes encoding *ampC*, *bla*_OXA-51_, and the efflux systems *adeB* and *abeM*.

## Discussion

The progressive and global spread of multidrug-resistant Gram-negative pathogens has led to increasingly difficult-to-treat nosocomial infections. Given the extremely limited treatment options currently available, novel therapeutic agents are urgently needed. Cefiderocol is a novel catechol-substituted siderophore cephalosporin with a broad spectrum of antimicrobial activity. Against *Enterobacterales*, MIC_90_ values of ∼0.5–1 mg/L have been reported, with >95% being inhibited by ≤4 mg/L (the proposed breakpoint for cefiderocol).^4,6,9,10^ Consistent with these reports, the *Enterobacterales* in our study had overall MIC_90_ values of 0.5 mg/L, and all were inhibited by ≤4 mg/L. In a study by Jacobs *et al.*, cefiderocol MICs were higher in *Enterobacterales* possessing an ESBL, carbapenemase, or AmpC-type β-lactamase compared with isolates lacking these enzymes.^12^ Our study also documented greater cefiderocol MICs in isolates of *E. coli* and *K. pneumoniae* that were cephalosporin resistant. The activity of cefiderocol has been reported to be maintained in *Enterobacterales* possessing a wide variety of carbapenemases, including KPC, NDM VIM, IMP, and OXA-48.^5,9,10,12^ The activity of cefiderocol was maintained in all our KPC-producing *K. pneumoniae*, with 90% of the isolates being inhibited by ≤2 mg/L, consistent with other reports.^10,12^

Cefiderocol has also been reported to have considerable activity against other Gram-negative pathogens. For *P. aeruginosa*, including carbapenem-resistant isolates, MIC_90_ values of 0.5–1 mg/L have been reported.^4–8,10,12^ In our study, 99.6% of our surveillance isolates had cefiderocol MICs ≤4 mg/L. MICs did not appear to be significantly affected by the development of cephalosporin or carbapenem resistance, consistent with findings reported elsewhere for *P. aeruginosa*.^12^ Similarly, for *A. baumannii* overall MIC_90_ values of 1–2 mg/L, and 8 mg/L for carbapenem-resistant isolates, have been reported.^4–8,10,12^ All our 2017 surveillance isolates, and 88% of our previous collection of carbapenem-resistant *A. baumannii*, were inhibited by ≤4 mg/L. For *A. baumannii*, cefiderocol MICs were unaffected by the presence of OXA-23, consistent with findings reported elsewhere.^10^ However, as with the *Enterobacterales*, we noted cefiderocol MICs were significantly higher in ESBL-possessing isolates of *A. baumannii*.

The catechol moiety in cefiderocol chelates free iron, enabling entry into bacteria through their iron transport system.^25^ As such, cefiderocol may evade porin-related bacterial mechanisms of antimicrobial resistance. Consistent with other reports, when our collection of characterized isolates of *K. pneumoniae* were examined, cefiderocol MICs were not significantly affected by alterations in the porins OmpK35 and OmpK36.^3,10^ In addition, we found no correlation between cefiderocol MICs and expression of several efflux-related genes. For *P. aeruginosa*, overexpression of the MexAB-OprM efflux system had no effect on the activity of cefiderocol.^3^ Similarly, in our collection of characterized isolates of *P. aeruginosa*, we found no correlation between expression of several efflux systems or in expression of the porin *oprD*. Finally, when tested against our collection of characterized isolates of *A. baumannii*, no correlation was found between cefiderocol MICs and expression of the efflux genes *adeB* and *abeM*.

Our study reaffirms the activity of cefiderocol against a large number of Gram-negative pathogens, including multidrug-resistant isolates. However, our findings may not be generalized to other multidrug-resistant pathogens, because only a limited variety of carbapenemases was identified (*bla*_KPC_ in *Enterobacterales* and *bla*_OXA-type_ in *A. baumannii*). Given the limited options available for many resistant nosocomial pathogens, our findings support the continued development of this agent.

## Supplementary Material

Supplemental data

Supplemental data

Supplemental data

## References

[1] Tacconelli E., Carrara E., Savoldi A., Harbarth S., Mendelson M.. Monnet D.L, Pulcini C., Kahlmeter G., Kluytmans J., Carmeli Y., Ouellette M., Outterson K., Patel J., Cavaleri M., Cox E.M, Houchens C.R, Grayson M.L, Hansen P., Singh N., Theuretzbacher U., Magrini N., and the Pathogens Priority List Working Group WHO 2018 Discovery, research, and development of new antibiotics: the WHO priority list of antibiotic-resistant bacteria and tuberculosis. Lancet Infect. Dis. 18:318–327 29276051 10.1016/S1473-3099(17)30753-3

[2] Bush K. 2015 A resurgence of β-lactamase inhibitor combinations effective against multidrug-resistant Gram-negative pathogens. Int. J. Antimicrob. Agents. 46:483–493 26498989 10.1016/j.ijantimicag.2015.08.011

[3] Ito A., Sato T., Ota M., Takemura M., Nishikawa T., Toba S., Kohira N., Miyagawa S., Ishibashi N., Matsumoto S., Nakamura R., Tsuji M., and Yamano Y. 2018 *In vitro* antibacterial properties of cefiderocol, a novel siderophore cephalosporin, against Gram-negative bacteria. Antimicrob. Agents Chemother. 62:e01454-17 10.1128/AAC.01454-17 PMC5740388 29061741

[4] Hackel M.A., Tsuji M., Yamano Y., Echols R., Karlowsky J.A, and Sahm D.F. 2017 *In vitro* activity of the siderophore cephalosporin, cefiderocol, against a recent collection of clinically relevant Gram-negative bacilli from North America and Europe, including carbapenem-nonsusceptible isolates (SIDERO-WT-2014 Study). Antimicrob. Agents Chemother. 61:e00093-17 28630181 10.1128/AAC.00093-17 PMC5571285

[5] Hackel M.A., Tsuji M., Yamano Y., Echols R., Karlowsky J.A, and Sahm D.F. 2018 *In vitro* activity of the siderophore cephalosporin, cefiderocol, against carbapenem-nonsusceptible and multidrug-resistant isolates of Gram-negative bacilli collected worldwide in 2014 to 2016. Antimicrob. Agents Chemother. 62:e01968-17 29158270 10.1128/AAC.01968-17 PMC5786755

[6] Karlowsky J.A., Hackel M.A, Tsuji M., Yamano Y., Echols R., and Sahm D.F. 2018 *In vitro* activity of cefiderocol, a siderophore cephalosporin, against Gram-negative bacilli isolated by clinical laboratories in North America and Europe in 2015–2016: SIDERO-WT-2015. Int. J. Antimicrob. Agents. 53:456–466 30471402 10.1016/j.ijantimicag.2018.11.007

[7] Hsueh S.C., Lee Y.J, Huang Y.T, Liao C.H, Tsuji M., and Hseuh P.R. 2019 *In vitro* activities of cefiderocol, ceftolozane/tazobactam, ceftazidime/avibactam and other comparative drugs against imipenem-resistant *Pseudomonas aeruginosa* and *Acinetobacter baumannii*, and *Stenotrophomonas maltophilia*, all associated with bloodstream infections in Taiwan. J. Antimicrob. Chemother. 74:380–386 30357343 10.1093/jac/dky425

[8] Ito A., Kohira N., Bouchillon S.K, West J., Rittenhouse S., Sader H.S, Rhomberg P.R, Jones R.N, Yoshizawa H., Nakamura R., Tsuji M., and Yamano Y. 2016 *In vitro* antimicrobial activity of S-649266, a catechol-substituted siderophore cephalosporin, when tested against non-fermenting Gram-negative bacteria. J. Antimicrob. Chemother. 71:670–677 26645269 10.1093/jac/dkv402

[9] Kohira N., West J., Ito A., Ito-Horiyama T., Nakamura R., Sato T., Rittenhouse S., Tsuji M., and Yamano Y. 2016 *In vitro* antimicrobial activity of a siderophore cephalosporin, S-649266, against Enterobacteriaceae clinical isolates, including carbapenem-resistant strains. Antimicrob. Agents Chemother. 60:729–734 26574013 10.1128/AAC.01695-15 PMC4750680

[10] Kazmierczak K.M., Tsuji M., Wise M.G, Hackel M., Yamano Y., Echols R., and Sahm D.F. 2019 *In vitro* activity of cefiderocol, a siderophore cephalosporin, against a recent collection of clinically relevant carbapenem-non-susceptible Gram-negative bacilli, including serine carbapenemase- and metallo-β-lactamase-producing isolates (SIDERO-WT-2014 Study). Int. J. Antimicrob. Agents. 53:177–184 30395986 10.1016/j.ijantimicag.2018.10.007

[11] Ito A., Nishikawa T., Ota M., Ito-Horiyama T., Ishibashi N., Sato T., Tsuji M., and Yamano Y. 2018 Stability and low induction propensity of cefiderocol against chromosomal AmpC β-lactamases of *Pseudomonas aeruginosa* and *Enterobacter cloacae*. J. Antimicrob. Chemother.73:3049–3052 30188999 10.1093/jac/dky317 PMC6198743

[12] Jacobs M.R., Abdelhamed A.M, Good C.E, Rhoads D.D, Hujer A.M, Domitrovic T.N, Rudin S.D, Richter S.S, van Duin D., Kreiswirth B.N, Greco C., Fouts D.E, and Bonomo R.A. 2019 ARGONAUT-I: activity of cefiderocol (S-649266), a siderophore cephalosporin, against Gram-negative bacteria, including carbapenem-resistant nonfermenters and Enterobacteriaceae with defined extended-spectrum β-lactamases and carbapenemases. Antimicrob. Agents Chemother. 63:e01801-18 30323050 10.1128/AAC.01801-18 PMC6325197

[13] Ito-Horiyama T., Ishii Y., Ito A., Sato T., Nakamura R., Fukuhara N., Tsuji M., Yamano Y., Yamaguchi K., and Tateda K. 2016 Stability of novel siderophore cephalosporin S-649266 against clinically relevant carbapenemases. Antimicrobial. Agents Chemother. 60:4384–4386 10.1128/AAC.03098-15 PMC4914688 27139465

[14] Portsmouth S., van Veenhuyzen D., Echols R., Machida M., Ferreira J.C.A, Ariyasu M., Tenke P., and Nagata T.D. 2018 Cefiderocol versus imipenem-cilastatin for the treatment of complicated urinary tract infections caused by Gram-negative uropathogens: a phase 2, randomised, double-blind, non-inferiority trial. Lancet Infect. Dis. 18:1319–1328 30509675 10.1016/S1473-3099(18)30554-1

[15] Iregui A., Ha K., Meleney K., Landman D., and Quale J. 2018 Carbapenemases in New York City: the continued decline of KPC-producing *Klebsiella pneumoniae*, but a new threat emerges. J. Antimicrob. Chemother. 73:2997–3000 30107491 10.1093/jac/dky322

[16] Abdallah M., Olafisoye O., Cortes C., Urban C., Landman D., Ghitan M., Collins B., Bratu S., and Quale J. 2016 Rise and fall of KPC-producing *Klebsiella pneumoniae* in New York City. J. Antimicrob. Chemother. 71:2945–2948 27353464 10.1093/jac/dkw242

[17] Landman D., Bratu S., and Quale J. 2009 Contribution of OmpK36 to carbapenem susceptibility in KPC-producing *Klebsiella pneumoniae*. J. Med. Microbiol. 58:1303–1308 19556371 10.1099/jmm.0.012575-0 PMC2887543

[18] Institut Pasteur. Institut Pasteur MLST and whole genome MLST databases. Available at http://bigsdb.web.pasteur.fr/index.html

[19] Abdallah M., Olafisoye O., Cortes C., Urban C., Charles C., Landman D., and Quale J. 2015 Reduction in the prevalence of carbapenem-resistant *Acinetobacter baumannii* and *Pseudomonas aeruginosa* in New York City. Am. J. Infect. Control. 43:650–652 25818022 10.1016/j.ajic.2015.02.018

[20] Quale J., Bratu S., Gupta J., and Landman D. 2006 Interplay of efflux system, *ampC*, and *oprD* expression in carbapenem resistance of *Pseudomonas aeruginosa* clinical isolates. Antimicrob. Agents Chemother. 50:1633–1641 16641429 10.1128/AAC.50.5.1633-1641.2006 PMC1472219

[21] Bratu S., Landman D., Martin D.A, Georgescu C., and Quale J. 2008 Correlation of antimicrobial resistance with β-lactamases, the OmpA-like porin, and efflux pumps in clinical isolates of *Acinetobacter baumannii* endemic to New York City. Antimicrob. Agents Chemother. 52:2999–3005 18591275 10.1128/AAC.01684-07 PMC2533509

[22] Hackel M.A., Tsuji M., Yamano Y., Echols R., Karlowsky J.A, and Sahm D.F. 2019 Reproducibility of broth microdilution MICs for the novel siderophore cephalosporin, cefiderocol, determined using iron-depleted cation-adjusted Mueller-Hinton broth. Diagn. Microbiol. Infect. Dis. 94:321–325 31029489 10.1016/j.diagmicrobio.2019.03.003

[23] Clinical and Laboratory Standards Institute. 2013 Methods for Dilution Antimicrobial Susceptibility Tests for Bacteria That Grow Aerobically- Ninth Edition: Approved Standard M07-A9. CLSI, Wayne, PA

[24] Clinical and Laboratory Standards Institute. 2018 Performance Standards for Antimicrobial Susceptibility Testing; 28th Informational Supplement, M100-S28. CLSI, Wayne, PA

[25] Ito A., Nishikawa T., Matsumoto S., Yoshizawa H., Sato T., Nakamura R., Tsuji M., and Yamano Y. 2016 Siderophore cephalosporin cefiderocol utiizes ferric iron transporter systems for antibacterial activity against *Pseudomonas aeruginosa*. Antimicrob. Agents Chemother. 60:7396–7401 27736756 10.1128/AAC.01405-16 PMC5119021

