# Supplementary material for: Activity of Cefiderocol Against Enterobacterales, Pseudomonas aeruginosa, and Acinetobacter baumannii Endemic to Medical Centers in New York City
Source: Microb Drug Resist. 2020 Jul 7;26(7):722–6. doi: 10.1089/mdr.2019.0298 (PMC7368386; doi:10.1089/mdr.2019.0298)
Supplement: Supplemental data [file Supp_TableS1.pdf]

Supplementary Data

Supplementary Table S1. Cefiderocol MICs and Data Associated with Antimicrobial Resistance in 34 Characterized Isolates of *Klebsiella pneumoniae*

	Cefiderocol	*KPC*	*ESBL*	bla_{KPC}	*acrB*	*ompK35*	*ompK36*
Isolate	*MIC (mg/L)*				*Relative expression*		
1	0.5	No	SHV		0.11	1.11	1.81
2	4		SHV		0.45	0.5	3.85
3	1	No	SHV		0.45	0.54	4.58
4	1	No	SHV		0.34	0.43	1.97
5	2	No	SHV		0.02	0.13	1.01
6	1	No	SHV		0	0.18	0.88
7	0.25	Yes	SHV	1	0.49	0.06	0.25
8	0.25	Yes	SHV	1.1	0.52	1.58	5.7
9	4	Yes	SHV	2.5	0.04	0.08	0.004
10	0.25	No	SHV		1.24	0.28	1.36
11	2	Yes	SHV	15	0.17	0.11	0.18
12	0.12	Yes	SHV	5	0.74	0.37	7.59
13	0.25	No	SHV		0.5	1.25	3.11
14	0.5	No	SHV		1.07	2.5	8.22
15	0.5	Yes	SHV	18.75	1.64	0.94	7.71
16	0.5	Yes	SHV	5	1.21	1.8	4.47
17	0.06	Yes	SHV	1.25	0.32	0.54	2.94
18	2	Yes	SHV	198.75	6.01	1.44	4.51
19	0.5	No	SHV		0.21	0.23	1.32
20	0.5	No			0.07	1.88	0.86
21	0.12	No			0.41	1.45	3.03
22	0.25	No			0.38	0.02	1.41
23	1	Yes	SHV	40	1.6	1.15	8.19
24	0.12	No			0.21	2.84	6.12
25	0.25	No			0.24	0.51	1.04
26	0.06				2.85	1.51	5.41
27	0.03				1.5	4.39	5.18
28	0.5				2.65	1.43	5.1
29	0.5	No			1.71	1.27	1.92
30	0.06	No			0.02	0.24	1.31
31	1	Yes		1.1	0.05	0.49	1.7
32	0.5	Yes		3.75	0.41	0.17	0.06
33	1	Yes		22.5	0.56	0.16	3.28
34	1	Yes		1	0.2	2.74	4.19
