# Supplementary material for: Activity of Cefiderocol Against Enterobacterales, Pseudomonas aeruginosa, and Acinetobacter baumannii Endemic to Medical Centers in New York City
Source: Microb Drug Resist. 2020 Jul 7;26(7):722–6. doi: 10.1089/mdr.2019.0298 (PMC7368386; doi:10.1089/mdr.2019.0298)
Supplement: Supplemental data [file Supp_TableS2.pdf]

Supplementary Table S2. Cefiderocol MICs and Expression of Genes Associated with Antimicrobial Resistance for 33 Isolates of *Pseudomonas aeruginosa*

Isolate	*Cefiderocol* MIC (mg/L)	bla_{ampC}	*oprD*	*mexA*	*mexC*	*mexE*	*mexX*
		Relative expression					
1	0.5	3.52	0	2.59	1.37	0.663	1.11
2	0.5	3.75	0	1	1.65	0.667	1.13
3	0.5	3.65	0	0.714	0.291	0.409	2.8
4	0.12	1.8	0	1.81	1.83	0.648	5.69
5	0.12	2646	0	1.93	0.173	0.05	29.4
6	0.12	2.4	0	1.65	0.151	0	31.2
7	1	2.08	0	1.04	1.03	0.228	34.6
8	0.12	1.28	0.024	0.961	0.671	0	9.62
9	0.06	483	0.035	0.523	0.069	0.196	6.69
10	1	928	0.0937	1.37	4.05	0.758	29.3
11	0.12	607	0.0954	0.411	0.077	0	24.3
12	0.12	269	0.132	1.13	0.867	0.236	31.6
13	1	376	0.135	0.872	0.196	0	87.7
14	4	65.5	0.164	0.721	0	0.009	0.951
15	0.5	2353	0.171	3.49	0.461	0.223	19.6
16	0.12	0.705	0.19	1.53	8.95	1.09	12.9
17	0.12	6.16	0.269	1.42	0	2.76	73.4
18	0.12	295	0.276	5.02	2.46	0.658	22.9
19	0.12	2.35	0.347	0.905	0.954	1.78	3.78
20	0.12	1.99	0.384	1.02	0.84	1.45	17.8
21	0.5	1469	0.464	3.12	2.02	0.6	42.6
22	0.5	169	0.561	0.879	2.86	0.414	52.6
23	0.5	974	0.705	2.83	0.171	0.006	19
24	0.12	29.2	0.711	3.48	1.79	0.191	0.06
25	0.12	2.62	0.813	0.97	2.69	0.395	5.03
26	0.5	7.47	0.943	1.45	4.36	0.713	4.4
27	0.12	3.96	1.02	1.75	9.43	1.69	4.01
28	0.25	2.67	1.23	2.41	2.21	0	5.7
29	0.25	1.36	1.79	1.67	0.767	1.22	6.64
30	0.12	5.06	2.36	12.6	9.3	1.64	331
31	0.5	0.31	4.06	3.94	3.04	0.427	5.82
32	0.12	0.412	10.1	2.22	16.1	0.546	16.2
33	0.06	5.46	11.1	2.21	2.13	0.211	6.87
