# Supplementary material for: Activity of Cefiderocol Against Enterobacterales, Pseudomonas aeruginosa, and Acinetobacter baumannii Endemic to Medical Centers in New York City
Source: Microb Drug Resist. 2020 Jul 7;26(7):722–6. doi: 10.1089/mdr.2019.0298 (PMC7368386; doi:10.1089/mdr.2019.0298)
Supplement: Supplemental data [file Supp_TableS3.pdf]

Supplementary Table S3. Cefiderocol MICs and Data Associated with Antimicrobial Resistance in 34 Characterized Isolates of *Acinetobacter baumannii*

Isolate	*Cefiderocol* MIC (mg/L)	bla_{ampC}	bla_{oxa51}	*adeB*	*abeM*	*ESBL*
		Relative expression				
1	4	0	6.25	4.2	1.6	SHV
2	0.25	0.12	0.76	0.08	1.2	
3	0.06	0.12	9.8	1.2	1.5	
4	0.12	0.18	119	1.3	1.6	
5	2	0.19	61.5	4.9	2.3	
6	0.12	0.25	36.2	3.2	2.4	
7	0.12	0.28	29.4	2	0.01	
8	0.12	0.49	10.2	5.4	1.8	
9	0.12	0.95	0.3	1	0.04	
10	4	4.9	26.1	1.4	1	SHV
11	4	5.2	296	0.67	1.1	SHV
12	0.12	7	163	0	1	
13	4	9.2	226	28.6	2	SHV
14	1	9.2	446	2.3	3.6	
15	32	12.3	17.5	1.3	2.7	SHV
16	4	14	58.6	2.2	1.8	SHV
17	2	75.7	30	27.3	4.2	SHV
18	1	83	26.6	2	1.3	
19	2	114	46.7	6.3	7.2	SHV
20	0.12	270	60	0.03	1.5	
21	1	401	23.1	14.3	2.1	SHV
22	1	10.4	388	4.4	8.5	
23	0.5	12	193	1.8	1.7	
24	2	16	189	1.2	2.2	SHV
25	8	16.6	1320	2.5	1.6	SHV
26	2	17.3	109	28.8	3.2	
27	0.12	17.6	405	0	0.78	SHV
28	4	18.3	2514	2.1	4.8	
29	0.5	35	160	1.5	2.7	SHV
30	1	44.4	2046	0.64	2	SHV
31	32	53	302	2.6	3.6	SHV
32	2	87	346	3.3	6.2	
33	32	169	404	3.4	0.92	SHV
34	4	248	1313	1.1	1.9	SHV
